# Comparison of the postoperative outcome with and without intraoperative leak testing for sleeve gastrectomy: a systematic review and meta-analysis of 469 588 cases

**DOI:** 10.1097/JS9.0000000000000919

**Published:** 2023-11-20

**Authors:** Longyin Ma, Zhenguo Gao, Heng Luo, Shien Kou, Yu Lei, Victor Jia, Ke Lan, Subbiah Sankar, Jiani Hu, Yunhong Tian

**Affiliations:** a Department of General Surgery, The Affiliated Nanchong Central Hospital of North Sichuan Medical College (University); b Department of Clinical Medicine, North Sichuan Medical College, Nanchong, Sichuan, People’s Republic of China; c School of Medicine, University of Michigan, Ann Arbor; d Department of Radiology, Wayne State University, Detroit, Michigan, USA

**Keywords:** intraoperative leak test, postoperative staple line leakage, prevention, sleeve gastrectomy

## Abstract

**Objective:**

Postoperative staple line leakage (SLL) after sleeve gastrectomy (SG) is a rare but serious complication. Many surgeons routinely test anastomosis with an intraoperative leak test (IOLT) as part of the SG procedure. This meta-analysis aims to determine whether an IOLT plays a role in reducing the rate of postoperative staple line related complications in patients who underwent SG.

**Methods:**

The authors searched the PubMed, Web of science, the Cochrane Library, and Clinical Trials.gov databases for clinical studies assessing the application of IOLT in SG. The primary endpoint was the development of postoperative SLL. Secondary endpoints included the postoperative bleeding, 30 days mortality rates, and 30 days readmission rates.

**Results:**

Six studies totaling 469 588 patients met the inclusion criteria. Our review found that the SLL rate was 0.38% (1221/ 324 264) in the IOLT group and 0.31% (453/ 145 324) in the no intraoperative leak test (NIOLT) group. Postoperative SLL decreased in the NIOLT group compared with the IOLT group (OR=1.27; 95% CI: 1.14–1.42, *P*=0.000). Postoperative bleeding was fewer in the IOLT group than that in the NIOLT group (OR 0.79; 95% CI: 0.72–0.87, *P*=0.000). There was no significant difference between the IOLT group and the NIOLT group regarding 30 days mortality rates and 30 days readmission rates (*P*>0.05).

**Conclusion:**

IOLT was correlated with an increase in SLL when included as a part of the SG procedure. However, IOLT was associated with a lower rate of postoperative bleeding. Thus, IOLT should be considered in SG in the situation of suspected postoperative bleeding.

## Introduction

As the prevalence of obesity has continued to increase worldwide, the number of performed bariatric procedures grows in parallel^1^. Among all bariatric procedures, laparoscopic sleeve gastrectomy (SG) is widely used worldwide in the surgical treatment of morbid obesity. It is considered to be a minimally invasive and safe surgery with low complications and mortality rates^2^. In SG, the stomach has its capacity reduced by approximately two-thirds^3^, which results in the patient eating less and losing weight^4^. Many advantages were shown in SG, such as reducing serum liver enzyme concentrations^5^, alleviating type 2 diabetes mellitus^5^, decreasing blood lipids^6^, and improving quality of life^7,8^ etc.

Intraoperative leak testing (IOLT) is a common intraoperative intervention to identify staple line leaks, defects, bleeding, and stricture. IOLT is often performed using air insufflation or methylene blue dye injection via upper gastrointestinal endoscopy or nasogastric tube^9^. Some studies recommend routine usage of the intraoperative leak test in SG^10–12^. However, the utility of these tests is controversial. The international SG expert panel failed to reach a consensus (48% consensus) about whether routine intraoperative leak tests should be performed^13^. A study showed that an IOLT using air insufflation or methylene blue dye was performed in 81.9% of cases and the leak rate was higher in patients with air insufflation or methylene blue versus without (0.8 vs 0.4%, *P*<0.01)^14^. In addition, IOLT has the possibility to cause iatrogenic injury due to excessive dilation of the remaining gastric pouch^15,16^.

To the best of our knowledge, this is the first meta-analysis regarding whether the IOLT procedure carries higher risk for postoperative staple line leakage. The aim of this study was to compare postoperative staple line leakage, postoperative bleeding, 30 days mortality rates, and 30 days readmission rates of IOLT with no intraoperative leak test (NIOLT) for SG.

## Methods

### Literature search strategy

The literature search for this systematic review was performed in January 2023 according to the Preferred Items for Reporting of Systematic Reviews and Meta-Analyses (PRISMA) guidelines^17^, and Assessing the methodological quality of systematic reviews(AMSTAR) Guidelines^18^. The study protocol was written and registered at The International Prospective Register of Systematic Reviews (Prospero) before data extraction. A systematic review of literature was performed by two authors independently using the databases PubMed, Web-of-Science, Cochrane Library, and Clinical Trials.gov databases along with a cross-reference search of eligible papers or trials. The following search strategy was used in PubMed and modified in other databases accordingly: ((sleeve gastrectomy) and (endoscopy) and (intraoperative) and (staple line leak)) or ((sleeve gastrectomy) and (stomach tube) and (intraoperative) and (staple line leak)) or ((sleeve gastrectomy) and (endoscopy) and (intraoperative leak testing)) or ((sleeve gastrectomy) and (stomach tube) and (intraoperative leak testing)) or ((bariatric surgery) and (stomach tube) and (intraoperative leak testing)) or ((bariatric surgery) and (endoscopy) and (intraoperative) and (staple line leak)) or ((bariatric surgery) and (stomach tube) and (intraoperative) and (staple line leak)) or ((bariatric surgery) and (stomach tube) and (intraoperative) and (staple line leak)) or ((endoscopy) and (intraoperative) and (staple line leak)) or ((stomach tube) and (intraoperative) and (staple line leak)) or ((endoscopy) and (intraoperative leak testing)) or ((stomach tube and (intraoperative leak testing)).

All studies comparing the postoperative outcomes of IOLT with NIOLT were included. Papers published before January 2023 were included. Moreover, we attempted to find all relevant literature by thoroughly looking through the references of included clinical articles. After analyzing the full texts, we identified a total of six studies that were suitable to be included in our meta-analysis.

### Study selection

Studies were included in the meta-analysis if they met the following criteria: 1) they conducted clinical trials comparing the postoperative outcomes of IOLT and NIOLT; 2) the study was published as a full-text in the English language; and 3) valid data and a full-text of the study could be obtained successfully.

### Study exclusion

Studies were excluded if they included patients that underwent any of the following procedures: mini-loop gastric bypass, endoscopic therapy, intragastric balloon, clinical trial, or experimental therapy. In addition, animal studies, conference abstract, comments, reviews, guidelines, and studies with fewer patients than 20 were excluded.

### Statistical extraction

Articles were first screened independently by two authors according to title and abstract, with disputes being resolved by a third author. This process was then repeated with a full-text review in which we extracted data including author, year of publication, country, study design, number of patients, sex, age, BMI, postoperative staple line leakage, postoperative bleeding, 30 days mortality rates, and 30 days readmission rates.

### Outcome

The primary endpoint was the development of postoperative staple line leakage. In this study, postoperative staple line leakage was defined as a leak after SG, which included intraoperative and postoperative finding leaks. Secondary endpoints included the postoperative bleeding, 30 days mortality rates, and 30 days readmission rates. The risk of bias was assessed using the Risk of Bias in Non-Randomized Studies of Intervention Tool^19^, which was shown in Table 1.

**Table 1:** Analysis for risk of bias of the studies included in the meta-analysis using the Risk of Bias in Non-Randomized Studies of Intervention Tool.

Studies	Baseline confounding	Selection of participant	Classification of diagnostic tools	Deviation from intended diagnostic	Missing data	Measurement of outcomes	Selection of reported results	Overall risk of bias
Binghan *et al*.^20^	Moderate	Low	Low	Low	Low	Low	Moderate	Moderate
Yolsuriyanwong *et al*.^21^	Moderate	Low	Low	Low	Low	Low	Low	Moderate
Sethi *et al*.^9^	Moderate	Low	Low	Low	Low	Moderate	Low	Moderate
Mayir *et al*.^22^	Moderate	Low	Low	Low	Low	Low	Low	Moderate
Jung *et al*.^23^	Moderate	Low	Low	Low	Low	Low	Low	Moderate
Liu *et al*.^24^	Moderate	Low	Low	Low	Low	Low	Moderate	Moderate

### Quality assessment

Quality assessment of the included studies was completed with the Newcastle–Ottawa Scale (NOS)^25^. Using this scale, each study was judged on eight items, categorized into three groups: the selection of the study groups; the comparability of the groups; and the exposure evaluation groups. Stars were awarded for each quality item and the highest quality studies were awarded up to nine stars. Scores of 7–9 points indicated high-quality studies, those of 4–6 points indicated moderate-quality studies, and those of 1–3 points indicated low-quality studies.

### Statistical analysis

For retrospective compared study, odds ratio (OR) was calculated. The Mantel–Haenszel method was used for dichotomous data, and the OR with 95% CIs was presented. To assess the significance in study heterogeneity, Cochran’s *P* statistic and *I*² were reported. If the data was found to be lacking in the published articles, authors were contacted for further inquiry. When heterogeneity was high, the random-effects model was used; otherwise, the fixed-effects model was used. Heterogeneity was explored using *I*² statistics and the analyses were illustrated with forest plots. Heterogeneity was calculated using the *I*² statistic and defined as low, moderate, and high when *I*² was more than 25, 50, and 75%, respectively^26^. We performed further subgroup analysis of the studies type of IOLT. Stata software (version 17.0; Stata Corporatio; College Station) was used to perform all analysis.

## Results

### Literature search results

**Figure 1:**
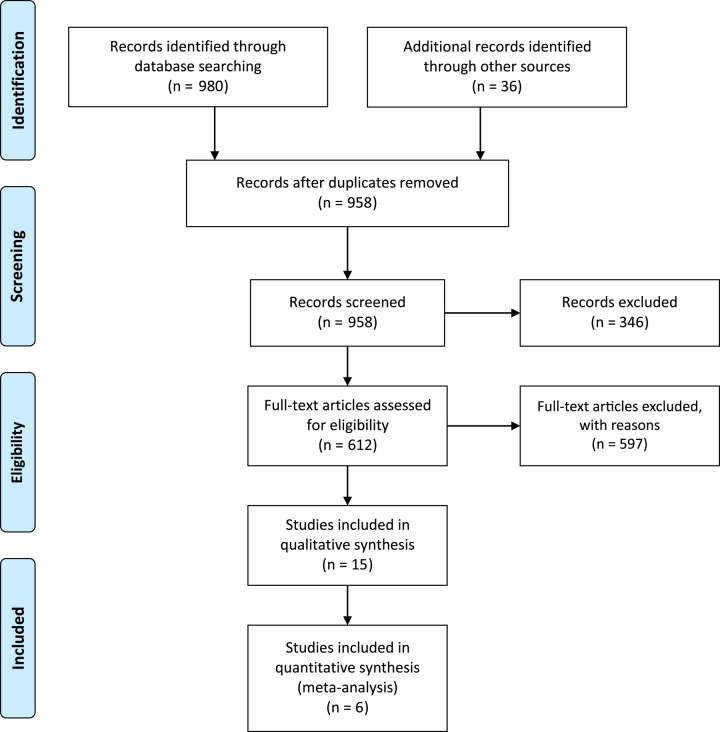
Flow diagram of study selection.

Our systematic search revealed a total of 1016 publications for possible inclusion. Based on a review of the title and abstract of each article, irrelevant publications, duplicate publications, and those not fitting our inclusion criteria were excluded. A further nine publications were excluded based on review of the full-text, leaving six retrospective studies that were included^9,20–24^ (Fig. 1).

### Study characteristics

The meta-analysis included 469 588 patients, of which 324 264 were assigned to the IOLT group and 145 324 to the NIOLT group. The studies were published between 2016 and 2022. One study originated from Turkey, while the other five originated from the United States. All studies performed intraoperative endoscopic or nonendoscopic methods (naso/orogastric tube insertion), which used air injection or used methylene blue to test for leakage. Details information on study characteristics are present in Table 2. Among the six studies in total, three of them reported positive results for IOLT^9,20,22^. Only two patients were reported as having a positive IOLT, which allowed for the reinforcement of the sutures. In the remaining three studies^21,23,24^, the IOLT group provided the leak rate after surgery instead of reporting positive results of IOLT. Details of distal clamp occlusion in IOLT were reported in three studies^9,21,22^, which showed in Table 3.

**Table 2:** The basic characteristics of included studies

Author	Country	Study period	Number of patients (IOLT/NIOLT)	Age (mean)	Sex (female/male)	BMI (kg/m^2^)	Type of study	Risk of Bias (NOS)☆	Type of intraoperative leak testing	Positive postoperative leak (IOLT/NIOLT)	Postoperative bleeding (IOLT/NIOLT)	30 days mortality rates (IOLT/NIOLT)	30 days readmission rates (IOLT/NIOLT)
Bingham *et al*.^[20]^	USA	2010–2014	2376/1908	45	3599/685	43	RS	7	AI or MBD	23/14	NR	NR	NR
Yolsuriyanwong *et al*.^[21]^	USA	2015–2016	142447/42091	45.3	NR	45.9	RS	8	MBD	354/96	NR	103/32	279/83
Sethi *et al*.^[9]^	USA	2012–2014	1329/221	40	1235/315	44.2	RS	8	AI or MBD	13/2	NR	0/1	NR
Mayir *et al*.^[22]^	Turkey	2017–2018	226/226	40	358/94	45.9	RS	8	MBD	2/2	3/3	NR	NR
Jung *et al*.^[23]^	USA	2015–2017	55405/55405	44	87484/23326	45	RS	8	AI or MBD	223/183	335/420	NR	1646/1711
Liu *et al*.^[24]^	USA	2015–2016	122481/45473	NR	NR	NR	ROS	7	EONAI or MBD	606/157	721/343	NR	3906/1463

**Table 3:** The details of distal clamp occlusion in intraoperative leak test.

Studies	Types of IOLT	Distal clamp occlusion
Sethi *et al*. ^9^	Air insufflation	Distal occlusion of the pylorus
	Methylene blue dye	Distal obstruction of the duodenum
Mayir *et al*. ^22^	Methylene blue dye	Pylorus was laparoscopically closed with a bowel clamp
Yolsuriyanwong *et al*.^21^	Methylene blue dye	The bowel was clamped distal to the anastomosis or staple line

IOLT, intraoperative leak test.

### Study quality

When using the NOS for case–control studies, the quality assessment of the included studies ranged from 6 to 8. All six studies had NOS quality scores greater than or equal to 6, indicating that all these studies had a high level of methodological quality. Table 4 shows the NOS quality scores of the included studies.

**Table 4:** Quality assessment of included studies.

	Selection					Outcome			
Author	Adequate case definition	Representative of cases	Selection of controls	Definition of controls	Comparability of cases and controls on basis of design of analysis	Ascertainment of exposure	Same Method of ascertainment for cases and controls	Nonresponse rate	Score
Binghan *et al*.^20^	☆	☆	☆	☆	☆	☆	☆		7
Yolsuriyanwong *et al*.^21^	☆	☆	☆	☆	☆	☆	☆		8
Sethi *et al*.^9^	☆	☆	☆	☆	☆	☆	☆		8
Mayir *et al*.^22^	☆	☆	☆	☆	☆	☆	☆		8
Jung *et al*.^23^	☆	☆	☆	☆	☆	☆	☆		8
Liu *et al*.^24^	☆	☆	☆	☆	☆	☆	☆		6

### Primary outcome: postoperative staple line leakage

**Figure 2:**
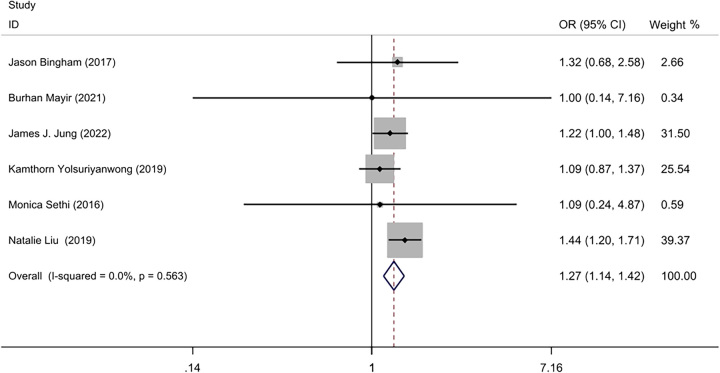
Forest plot of postoperative staple line leakage.

Our review^9,20–24^ found that the SLL rate was 0.38% (1221/ 324 264) in the IOLT group, and 0.31% (453/ 145 324) in the NIOLT group. A low statistical heterogeneity was detected between the six studies (*I*²=0%, *P*=0.56), so a fixed effect model was used for meta-analysis. The meta-analysis showed that postoperative staple line leakage was lower in the NIOLT group than the IOLT group (OR=1.27; 95% CI: 1.14–1.42, *P*=0.000) (Fig. 2).

### Subgroup analysis

**Figure 3:**
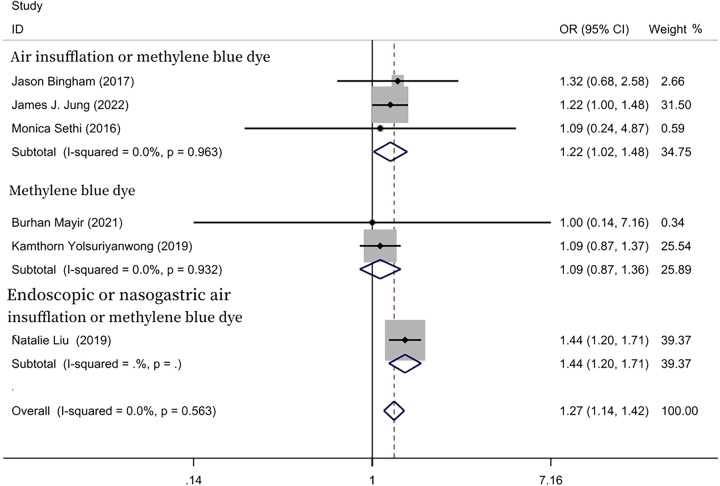
Forest plot of subgroup analysis of leaking test type.

We performed further subgroup analysis of the included studies, which was done accordance with the method of every study of IOLT. In two included studies, the methylene blue test was adopted in the IOLT group^21,22^. The postoperative staple line leakage rate was 0.25% (356/142 673) in the IOLT group, and 0.23% (98/42 317) in the NIOLT group. The meta-analysis showed no statistically significant differences in the IOLT group and in the NIOLT group (OR=1.09; 95% CI: 0.87–1.36, *P*=0.458). However, in three included studies, air insufflation or methylene blue dye were adopted in the IOLT group^9,20,23^. The postoperative staple line leakage rate was 0.44% (259/59 110) in the IOLT group, and 0.34% (198/57 534) in the NIOLT group. The postoperative staple line leakage was lower in the NIOLT group than that in the IOLT group (OR=1.22; 95% CI: 1.02–1.48, *P*=0.033). Only one study was suitable for meta-analysis, which used endoscopic or nasogastric air insufflation or methylene blue in the IOLT group^24^ (Fig. 3).

### Secondary outcomes

#### Postoperative bleeding

**Figure 4:**
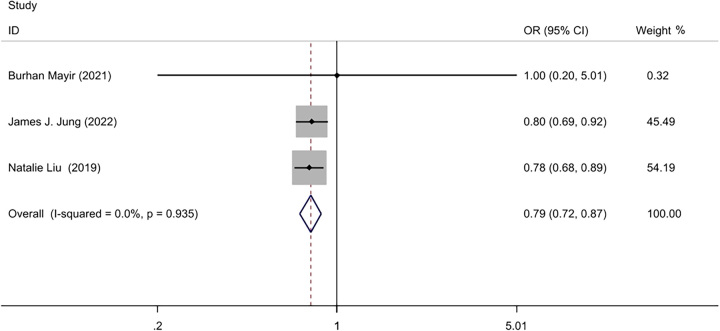
Forest plot of postoperative bleeding.

Three studies^22–24^ for a total of 279 216 patients reported postoperative bleeding. The postoperative bleeding rate was 0.59% (1059/178 112) in the IOLT group, and 0.76% (766/101 104) in the NIOLT group. A Fixed-effect model was used with low statistical heterogeneity (*I*^2^=0, *P*=0.94). The meta-analysis showed that postoperative bleeding was lower in the IOLT group than that in the NIOLT group (OR=0.79; 95% CI: 0.72–0.87, *P*=0.000) (Fig. 4).

#### 30 days mortality rates

**Figure 5:**
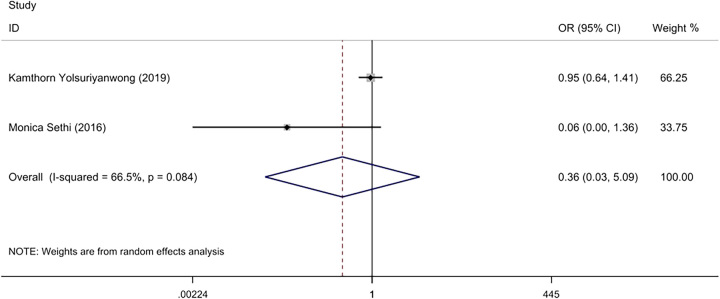
Forest plot of 30 days mortality rates.

Two of the included studies^9,21^ reported on the 30-day mortality rates of patients. The mortality rate was 0.1% (103/143 776) in the IOLT group, and 0.1% (33/42 312) in the NIOLT group. Due to the moderate heterogeneity (*I*^2^=66%, *P*=0.08), a random-effect model was used for meta-analysis, which found no statistically significant differences in the 30-day mortality rates between the two groups (OR=0.36; 95% CI: 0.03–5.09, *P*=0.45) (Fig. 5).

#### 30 days readmission rates

**Figure 6:**
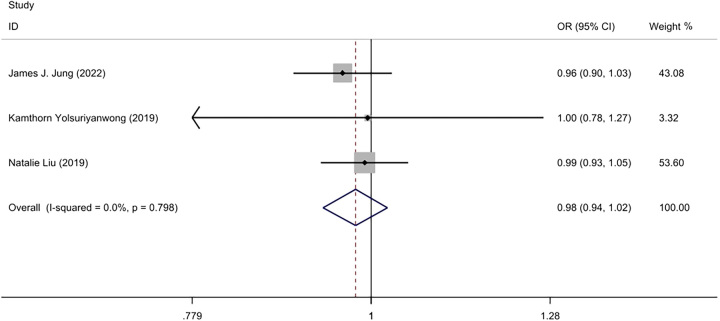
Forest plot of 30 days readmission rates.

1.78% (5700/320,333) patients had 30 days readmission rates in the IOLT group, and 2.28% (1,794/142 996) in the NIOLT group^21,23,24^. Due to the low statistical heterogeneity (*I*²=0%, *P*=0.79), a Fixed-effect model was used. Analysis determined that there was no significant difference in the 30 days readmission rates between the IOLT group and the NIOLT group (OR=0.98; 95% CI: 0.94–1.02, *P*=0.33) (Fig. 6).

## Discussion

SG, also known as vertical SG or gastric sleeve, was initially described in 1988^27^ and is a commonly performed surgery for weight loss^3^. Postoperative SLL is one of the most severe complications following SG^13^. The incidence of SLL after SG is relatively low, with a reported incidence ranging from 0 to 8%^28–30^. To the best of our knowledge, this is the first meta-analysis regarding whether the IOLT procedure carries higher risk for postoperative staple line leakage.

In this study, we observed that NIOLT has a lower rate of postoperative staple line leakage compared to IOLT. One possible explanation for this is that postoperative leakage may occur due to a fault in the testing mechanism. For example, the calibration tube is already present in the stomach before stapling. When the test is about to be conducted, the calibration tube is gradually drawn up to the upper stomach, and then the test is performed. Therefore, there may be no need to insert it, thus reducing the risk of staple line injury.

A few different techniques for IOLT have been reported^9,31^. In a study by Burgos AM, IOLT involved administering methylene blue through a nasogastric tube placed after the removal of the bougie, with the goal of protecting against suture-line leaks and aiding in the evaluation of gastric capacity^9^. In another study by Sethi M, IOLT included methylene blue testing upon completing the sleeve, followed by the removal of the bougie and the placement of an orogastric or nasogastric tube under direct vision^9^. Another potential explanation for the higher leak rate is that many surgeons employ the orogastric tube method for IOLT^31^. This method, due to its blind insertion nature, can potentially cause trauma to the freshly constructed staple line, possibly leading to postoperative leaks. Additionally, the pressures exerted during the air insufflation leak test can weaken the staple line, increasing the risk of postoperative leak development^32^.

IOLT is safe and effective in gastric bypass surgery^33–35^. The following reasons can explain the increased leakage rate of IOLT in SG in specific. First, Roux-en-Y gastric bypass is a more complicated procedure with multiple anastomoses, making it harder to completely visualize the anastomosis, particularly on the posterior side^36^. Compared to gastric bypass, SG involves a simpler linear stapling. Therefore, the data supporting IOLT during Roux-en-Y gastric bypass cannot necessarily be extrapolated to SG^20^. Second, SG, in comparison to gastric bypass, retains the intact pylorus and has higher intraluminal pressure, which may lead to an increased leak rate^28^.

Our findings are in accordance with recent studies that indicated increased postoperative leak rates when IOLT was performed in SG^14,24,37^. A consensus report published in the Netherlands showed that IOLT was not considered to be a key step in SG^38^. A 494 patients study showed that the routine use of an IOLT did not reduce the incidence of postoperative leak, and in fact was associated with a higher leak rate after SG^37^. Furthermore, the usage of air insufflation or methylene blue dye tests were not completely advantageous and could add operative times and unnecessary costs to the surgery^9^. In addition, a negative methylene blue test does not eliminate the possibility of a leak^39^. Some studies showed that routine tests to rule out leaks seem to be superfluous^40,41^. However, some studies showed that an intraoperative leak test was an effective method for detecting leakage after SG^10–12^. Other studies found that performing IOLT was not associated with postoperative leak in patients who underwent SG^20,21,37,42^.

There are patients experiencing leak postoperative even though IOLT was negative. Some possible explanations are as follows. First, IOLT can only detect the rare leaks due to technical failure in the staple line, such as stapler misfire^40^. Second, it has been reported that IOLT has a low sensitivity and specificity, which does not result in decreased postoperative leak rates after SG^20^. A study showed that upper gastrointestinal radiography found leaks, but the IOLT result was negative^20^.

We found that the postoperative bleeding was significantly lower in the IOLT group. Our findings are in align with recent studies indicating that IOLT during for SG was associated with lower rates of postoperative bleeding (0.6 with leak testing versus 0.8%; *P*<0.001)^24^. An observational cohort study has also demonstrated that patients who had underwent IOLT during SG had lower postoperative bleeding rates^21^. Furthermore, a separate study has shown that IOLT was associated with a decrease in postoperative bleeding rates in SG patients (0.6 with IOLT versus 0.8% without IOLT, *P*=0.002)^23^. One possible explanation for this is that the potential postoperative bleeding, when detected through IOLT, allows surgeons to promptly implement hemostatic measures^23^. It has been reported that leak testing may be justified in cases of revisional surgery, intraoperative complications, or in the case of a surgeon who is in the learning curve stage^9^. Thus, we suggest that IOLT should be considered in SG in the situation of suspected postoperative bleeding.

Additionally, our study showed that regardless of whether patients had received IOLT or not, the rates of postoperative 30 days mortality rates and 30 days readmission rates were not significantly different(*P*>0.05). Our findings are in accordance with recent studies indicating that there were no significant differences between the IOLT and NIOLT groups in terms of 30 days mortality and 30 days readmission rates^21^. In addition, a study found that performing IOLT was not associated with changes in rates of 30 days readmission rates^23^.

Our study has some limitations. First, all included studies were conducted retrospectively, which may introduce selection bias and potentially reduce the reproducibility of our results. Second, the technique of IOLT was not standardized; some studies employed endoscopy with methylene blue and air tests for IOLT while others used only an orogastric tube with methylene blue or air tests. Additionally, the pressure of IOLT in the remnant of gastric sleeve was not monitored. Third, only three studies reported the positive cases of IOLT, making it difficult to calculate the salvage rates, which would have allowed reinforcing the staple line when intraoperative leaks were detected.

In conclusion, IOLT was correlated with an increase in staple line leakage. However, IOLT was associated with a lower rate of postoperative bleeding. Prospective studies proposing a systematic way of performing IOLT are still needed. Further studies, perhaps incorporating manometric factors into IOLT, should be considered.

## Ethics approval and consent to participate

All procedures followed were in accordance with the ethical standards of the ethics committee of Nanchong Central Hospital and with the Helsinki Declaration of 1964 and later versions. Informed consent was obtained from all patients.

## Consent for publication

Written informed consents were obtained from the patients for publication of the two case reports and any accompanying images.

## Sources of funding

This work was supposed by the Foundation of Sichuan Medical Association [S21025], the Cooperative project of Nanchong City with North Sichuan Medical College [20SXQT0321], and the Bureau of Science and Technology Nanchong City [22JCYJPT0007].

## Author contribution

Y.T., L.M., and Z.G.: have made substantial contributions to the design of the work; H.L. and J.H.: interpreted the patients’ data; L.M., L.Y., and V.J.: was a major contributor in writing the manuscript; Y.T., S.K., K.L., and S.S.: have drafted the work or substantively revised it. All authors read and approved the final manuscript.

## Conflicts of interest disclosure

The authors declare that they have no competing interest associated with this manuscript.

## Research registration unique identifying number (UIN)

The International Prospective Register of Systematic Reviews (Prospero) (CRD:42023393776).

## Guarantor

Yunhong Tian.

## Data availability statement

All the data in this study are truly reliable and were done in collaboration with all the authors.

## Provenance and peer review

Not commissioned, externally peer-reviewed.
